# Rate and Associated Factors of Suicidal Behavior among Adolescents in Bangladesh and Indonesia: Global School‐Based Student Health Survey Data Analysis

**DOI:** 10.1155/2022/8625345

**Published:** 2022-08-05

**Authors:** M. Marthoenis, S. M. Yasir Arafat

**Affiliations:** ^1^Department of Psychiatry and Mental Health Nursing, Universitas Syiah Kuala, Banda Aceh 23111, Indonesia; ^2^Department of Psychiatry, Enam Medical College and Hospital, Dhaka-1340, Bangladesh

## Abstract

**Objective:**

Suicidal behavior among adolescents is a major public health problem that is understudied in South East Asian Muslim-majority countries. We aimed to investigate the rate and associated factors of suicidal behavior among adolescents in Bangladesh and Indonesia.

**Methods:**

The Global School-based Student Health Survey data of Bangladesh and Indonesia were used in this study. The data consist of a total of 9052 school-aged students from Bangladesh (2570, 28.4%) and Indonesia (6482, 71.6%). Suicidal behavior was assessed using three questions that measure suicidal ideation, suicidal plan, and suicidal attempts.

**Results:**

The overall rate of suicidal behavior (suicidal ideation, suicidal plan, and suicidal attempts) was 8.8%, and no significant difference between the two countries (8.9% in Bangladesh and 8.7 in Indonesia) was observed (*p*=0.81). Factors that independently increased the likelihood of suicidal behavior include female gender, missed class, physical fight four times or more, experienced bullying, anxiety, loneliness, rarely eating fruit, current alcohol use, and sedentary behavior (*p* < 0.05). Meanwhile, factors that independently decreased the likelihood of suicidal behavior include parental supervision and having close friends, either one, two, three persons, or more (*p* < 0.05).

**Conclusion:**

The study revealed rates and risk factors of suicidal behaviors among the school-going adolescents of two Muslim-majority countries in South East Asia. Prevention strategies should be considered guided by the risk factors for school-going adolescents.

## 1. Introduction

Suicide is a global public health problem across the globe and the ages of life [1, 2]. It is an endpoint of a complex interaction between personal, social, cultural, and religious vulnerability factors and is the second leading cause of death among adolescents [1]. As per the World Health Organization (WHO) report, nearly four of five global suicides occur in low-income and middle-income countries (LMICs) [3], where suicidal behavior remains underexplored.

Suicidal behavior is a common term used to address suicide-related conduct, either fatal or nonfatal. Nonfatal suicidal behavior (suicidal behavior) refers to the act that does not result in death and this includes suicidal ideation, plan, and attempt [4, 5]. Furthermore, several theories have been developed to try to explain the cause and the process of suicidal behavior [6, 7]. The interpersonal theory of suicide for instance proposed that suicidal desire is triggered by the simultaneous presence of perceived burdensome and thwarted belongingness [7]. The three-step theory, on the other hand, proposed that any suicidal behavior should pass three steps, feeling pain and hopelessness, idea escalation, and lastly the capacity to commit suicide [6]. These theoretical proposals, after all, required further investigations.

The suicide rate is lower in the Muslim-majority countries in comparison to other religions albeit several issues have been noted such as criminal status, stigma, and underreporting [8–10]. However, in the case of suicidal behavior, the rate is variable as evident by an earlier study among university students in 12 Muslim-majority countries that found a wide range of rates of suicide ideation and suicide attempt. The lowest rate of suicide ideation was found in Malaysia (0.9%) and the highest in Saudi Arabia (38.7%). Meanwhile, the lowest rate of suicide attempts was also reported in Malaysia (0.9%) and the highest rate was in Azerbaijan (20.5%) [11]. The diversities in regards to the practice of Islam, ethnicity, social, cultural, and economic status among the Muslim majority countries [9, 12] could be responsible for the suicidal behavior of Muslims. An earlier report among the orthodox majority in Ethiopia suggests that the lifetime prevalence of suicide ideation, suicide plan, and suicide attempt in adolescents was 22.5%, 24%, and 16.2%, respectively [13]. Meanwhile, among adolescents in Thailand, the Buddhist-majority country, the suicide ideation rate during the past 12 months was 8.8% [14]. A study in India, a Hindu-majority country, found a prevalence of suicide ideation of 30.9% [15].

Earlier studies highlighted various factors associated with suicidal behavior among adolescents [11, 13–20]. The factors include dissatisfied grade results, school truancy, loneliness, hopelessness, poor social support, physically attacked, alcohol use [12], poor parental attachment, sexual intercourse [14], female gender, food insecurity [16], and bullying [17]. Two large studies that assessed the suicidal behaviors among university students in Muslim-majority countries [11, 18] have also been reported. South East Asia (SEA) is a densely populated region that caters to 26% of the global population and contributes to 39% of global suicides [1, 21]. Suicide prevention targeting adolescents in this region, however, remains inadequate. Using the GSHS data, the suicidal behavior among adolescents in Bangladesh [19] and Indonesia [20] has been separately reported. Therefore, in this study, we aimed to investigate the rate and associated factors of suicidal behavior among adolescents in the two Muslim-majority countries of SEA, i.e., Bangladesh and Indonesia. The research questions were as follows: what is the prevalence of suicidal behavior among the adolescents of Bangladesh and Indonesia? What are the factors independently associated with suicidal behavior among them? The suicidal behavior in this report includes suicidal ideation, suicidal plan, and suicidal attempt. The findings of the study would help find out the homogeneous risk and protective factors based on religion in SEA that could be helpful while considering the prevention strategies.

## 2. Methods

### 2.1. Data Source

The data were extracted from the WHO Global School-based Student Health Survey (GSHS), which was designed to collect data from school-aged adolescents in developing countries, including Bangladesh and Indonesia [22]. Data collection was conducted in 2014 in Bangladesh and 2015 in Indonesia. A two-stage cluster sample design was used to collect data from students' classes 7 to 12 in Indonesia [23] and classes 7 to 10 in Bangladesh [24]. At the first stage, schools were chosen with a probability proportional to enrolment size. At the subsequent stage, random sampling methods were used to select the class in each school, and all students selected in each class were eligible to participate in the study. The pooled data from Bangladesh and Indonesia were used in order to unveil the associated factors of suicidal behavior in these two SEA Muslim-majority countries. The student's response rate in both Bangladesh and Indonesia was 94%, with a total of 11,142 students in Indonesia participating in the study [23] and 2989 students in Bangladesh [24]. After data cleaning by deleting respondents with empty responses, a total of 9052 respondents were included in this report consisting of 2570 (28.4%) from Bangladesh and 6482 (71.6%) from Indonesia. The detailed explanation of the survey from both countries is available at https://www.cdc.gov/gshs/countries/seasian/index.htm.

### 2.2. Measures

A self-administered questionnaire to obtain data among adolescents was used in the survey. The questionnaire contains questions on the adolescents' alcohol use, dietary behavior, drug use, personal hygiene, physical activity, protective factors, sexual behavior, tobacco use, violence and injury, and their mental health, including suicidal behaviors. The questionnaire, which includes the questions, response options, and coding used in this study, is presented in Supplementary file 1.

### 2.3. Outcome Variables

Suicidal behavior was assessed using three questions that measure suicidal ideation, suicidal plan, and suicidal attempts in adolescents. The GSHS measures suicidal ideation using the question “during the past 12 months, did you ever seriously consider attempting suicide?” For the suicidal plan, the question was “during the past 12 months, did you make a plan about how you would attempt suicide?” For both questions, the response option was “yes” and “no.” The suicidal attempt was assessed using the question “during the past 12 months, how many times did you actually attempt suicide?”, with the response option “0 (never),” “1,” “2 or 3,” “4 or 5,” and “6 or more” times. The question on suicide attempt was recoded into “no attempt” and “attempted”. The binary variable of suicidal behavior was created from these three questions, where any “yes” answer from them is considered as having “suicidal behavior.”

### 2.4. Covariate

Sociodemographic variables include age and gender. Psychosocial factors include missing class, the experience of bullying, and being famished in the past 30 days, anxiety, loneliness, physically attacked, and physical fight in the last 12 months. Healthrisk behaviors include not eating fruit, cigarette use and alcohol use in the past 30 days, ever being drunk from alcohol, having trouble from using alcohol, ever using amphetamine, and sedentary behavior. Protective factors include parental supervision, parental emotional support, parents know free time, and the number of close friends.

### 2.5. Data Analysis

Data were described using frequency and percentage. The differences in proportion between variables were calculated using the Pearson chi-square test. Simple logistic analysis was used to calculate the crude odds ratio (OR) of covariate variables toward suicidal behavior. Multivariate analysis was employed to calculate the adjusted odds ratio (AOR) for variables with *p* value <0.2 during bivariate analysis. The AOR was adjusted to other associated variables in bivariate analysis. *p* value of <0.05 was considered statistically significant during multivariate analysis. Data were analyzed using the STATA statistical software [25].

### 2.6. Ethical Approval

No institutional approval was sought as we used publicly available secondary data. We fully complied with the Declaration of Helsinki.

## 3. Results

### 3.1. Rate of Suicidal Behavior

The age ranged from 11 years or less to 18 years or more, and almost half of them (48.7%) aged between 14 and 15 years . More than half (58.4%) of the subjects were female. The overall rate of suicidal behavior in both countries was 8.8%. No significant difference in the suicidal behavior rate between countries was observed (8.9% in Bangladesh and 8.7 in Indonesia), (*x*^2^ = 0.05, *p*=0.81). The rate of suicide ideation, suicide plan, and suicide attempt during the past 12 months in both countries was 4.9%, 5.6%, and 3.1%, respectively. Detail of suicidal behavior rates in both countries is presented in Table 1.

### 3.2. Risk Factors

Compared to the overall rate, the rate of suicidal behavior was statistically higher among those whose gender was female (9.7%), who missed class 1-2 days (10.2%) or 2 days or more (17.6%) in the past 30 days, who were physically attacked in the past 12 months (2 or 3 times = 11.2% and 4 times or more = 15.2%), who were involved in a physical fight in the past 12 months (2 or 3 times = 11.8% and 4 times or more = 21.3%), who experienced bullying in the past 30 days (1 or 2 days = 12.5% and 3 or more days = 22.5%), who had been most of the time or always so worried about something in the past 12 months (24.9%), who felt lonely (23.5%), and who were famished because of having no food at home in the past 30 days (11.4%) (*p* < 0.05). Details of suicidal behavior rates of each sociodemographic characteristics, health risk, and protective factors are presented in Table 2.

Adolescents engaged with health-risk behaviors had also a higher rate of suicidal behavior. The rate was higher among those who do not eat any fruit (12%), smoke cigarettes (10.5%), and use alcohol (20.1%) in the past 30 days. The rate was also higher among those who in their lifetime ever drunk from using alcohol (17.8%) and use amphetamine (35.9%). Details of suicidal behavior rates of each sociodemographic characteristics, health risk, and protective factors are presented in Table 2.

Logistic regression analysis confirms several factors that are independently associated with suicidal behavior. Being a female was 1.6 times more likely to exhibit suicidal behavior than being a male (95% CI: 1.33–1.86) (Table 3). Those who missed class for 1 or 2 days were 1.3 times more likely to have suicidal behavior (95% CI: 1.08–1.55), and those who missed 3 or more days were 1.7 times more likely to have suicidal behavior (95% CI: 1.21–2.33). Adolescents involved in a physical fight 4 times or more in the past 12 months were 1.9 times more likely to have suicidal behavior (95% CI: 1.35–2.76), while those who were physically attacked 4 times or more in the past 12 months were 1.3 times more likely to have suicidal behavior (95% CI: 1.02–1.74). Adolescents who in the past month experienced bullying 1 or 2 more days were 1.4 times more likely to have suicidal behavior (95% CI: 1.15–1.73), while those who experienced it 3 or more days were 2.4 times more likely to have suicidal behavior than those who had never been bullied (95% CI: 1.82–3.06). Furthermore, adolescents who had been so worried about something that they could not sleep during the night were 2.04 more likely to have suicidal behavior (95% CI: 1.53–2.73), while those who felt loneliness during the past 12 months were 2.16 more likely to have suicidal behavior (95% CI: 1.71–2.73). Adolescents who rarely ate fruit during the past month were 1.33 times more likely to have suicidal behavior (95% CI: 1.04–1.70), while those who actively drank alcohol were 1.86 more likely to have suicidal behavior (95% CI: 1.17–2.96), and those who exhibited sedentary behavior were 1.24 more likely to have suicidal behavior (95% CI: 1.04–1.48).

### 3.3. Protective factors

The rate was lower among adolescents with good parental supervision (6.6%), those parents who respect adolescents' free time (7.7%), and those who have 3 or more close friends (7.6%) than those who have no close friends at all (24.1%). Further analyses confirm several protective factors against suicide, which include parental supervision (AOR = 0.62, 95% CI: 0.52–0.74) and having close friends one person (AOR = 0.35, 95% CI: 0.25–0.48), two persons (AOR = 0.38, 95% CI: 0.27–0.53), and 3 or more person (AOR = 0.28, 95% CI: 0.22–0.37).

## 4. Discussion

This study found that the rate of suicide ideation, suicide plan, and suicide attempt among adolescents in two Muslim-majority countries during the past 12 months was 4.9%, 5.6%, and 3.1%, respectively. The rates have been significantly lower than reported in other non-Muslim-majority countries [13–15]. The lower rate of suicidal behavior in Muslim-majority countries has been widely noticed. In fact, practicing Islam even offer protection against suicide [26]. There are several explanations for the lower rate of suicide in Muslim society. The concept of life in Islam is against suicidal behavior. Suicide is a big sin and completely forbidden. Islam establishes that only the creator has absolute power over human life, and any act of killing and hurting the creations is forbidden [27]. Thus, practicing Muslims are more likely to reject any suicidal behavior and have better gratitude, optimism, mindfulness, emotional regulation, and patients than non-religious groups [26]. Adolescents in Muslim-majority countries have been taught by their parents or religious teachers about these beliefs and attitudes since their younger age. This religious education seems to be protective against suicidal behavior in their life. However, variations should have been expected from country to country (between Muslim-majority countries), region to region (Muslim-majority country between Asia and Africa), and behavior to behavior (idea, plan, and attempt) as suicidal behavior is a complex phenomenon with intricate interactions among sociocultural factors [5, 11, 12, 18]. One large-scale recent review also identified such variations [11]. Further studies are warranted to explore the differences.

The finding of a higher rate of suicide ideation and plan compared to suicide attempts among adolescents in this study has been consistent with the situation in other low-income countries [28].

This study also found several factors that are independently associated with suicidal behavior. It revealed that school truancy, being involved in physical fight, alcohol use, female gender, and bullying victims are associated with suicidal behvaior [13, 16]. This study adds that having sleep problems, rarely eating fruit, and being engaged with sedentary behavior are the factors that increase the likelihood of suicide. It is unclear how sleeping, eating, and sedentary behavior contribute to suicidal behavior. Considering sleep problem, poor eating, and sedentary behavior as the signs of depression might explain their contribution to suicidal behavior. Furthermore, obtaining sufficient parental support and having close friends are found to be protective against suicide. This finding is in line with earlier reports, where parental support was negatively associated with suicidal thoughts [29] and perceived lower parental support independently predicted suicide attempts [30]. Having close friends is considered to be a protective factor against suicidal behavior [16, 31]. For adolescents, having parental support and close friends means that they have people around with whom they can share their problems. It is therefore encouraged for parents to provide more adequate support to their adolescents and the school authorities to organize such activities where students could maintain or extend their friendship with other students.

### 4.1. Strength of the Study

To the best of the authors' knowledge, this is the first study assessing the suicidal behaviors in two Muslim-majority countries of a suicide-dense region. This study also includes attention of antecedents (risk and protective factors) for adolescent suicidal thought and behavior in two SEA Muslim-majority countries. The findings of the study would help assess the impact of religious belief on suicidal behavior of adolescents in SEA.

### 4.2. Limitations of the Study

The first limitation of the study is that we used data from two different years for two different countries, i.e., 2014 for Bangladesh and 2015 for Indonesia. Data were collected more than five years ago. We considered the religion of the population of the country instead of the religion of the respondents. The second limitation is that the GSHS survey only collected data on age and gender for the sociodemographic factors. Meanwhile, various studies show the significant contribution of socioeconomic conditions toward suicidal behavior. Lastly, the assessment outcome and other variables were based on self-report by participants with no mean of response validation. The adolescents might have misunderstood the question and are therefore prone to response bias.

## 5. Conclusion

The study revealed rates and risk factors of suicidal behaviors among the school-going adolescents of two Muslim-majority countries in South East Asia. Prevention strategies should be considered guided by the risk factors while formulating the suicide prevention guidelines considering religion as a homogeneous factor between the countries. Further studies are warranted to assess the factors of having similar rates in two different countries.

## Figures and Tables

**Table 1:** The prevalence of suicidal behavior among adolescents in Bangladesh and Indonesia.

Countries	Sample size	Suicide ideation	Suicide plan	Suicide attempt	Suicidal behavior
	*n* (%)	*n* (%)	*n* (%)	*n* (%)	*n* (%)
Bangladesh	2570 (28.4)	107 (4.2)	138 (5.4)	106 (4.2)	228 (8.9)
Indonesia	6482 (71.6)	334 (5.2)	364 (5.6)	172 (2.7)	565 (8.7)
Total	9052 (100)	441 (4.9)	502 (5.6)	278 (3.1)	793 (8.8)

**Table 2:** Demographic characteristics and their associations with suicidal behavior (*n* = 9052).

Variables	Total sample *n* (%)	Suicidal behavior *n* (%)	*p* value (*X*^2^)
Sociodemographic			
Age group in year			>0.05
13 or less	3029 (33.5)	252 (8.3)	
14-15	4408 (48.7)	392 (8.9)	
16 or more	1615 (17.8)	149 (9.2)	
Gender			*p* < 0.0001
Male	3766 (41.6)	282 (7.5)	
Female	5286 (58.4)	511 (9.7)	

*Psychosocial factors*			
Missed class			*p* < 0.0001
Never (0 days)	6775 (74.8)	535 (7.9)	
1 or 2 days	1936 (21.4)	198 (10.2)	
3 or more days	341 (3.8)	60 (17.6)	
Physically attacked			*p* < 0.0001
Never (0 times)	5747 (63.5)	417 (7.3)	
1 time	1258 (13.9)	116 (9.2)	
2 or 3 times	1284 (14.2)	144 (11.2)	
4 times or more	763 (8.4)	116 (15.2)	
Physical fight			*p* < 0.0001
Never (0 times)	7273 (80.3)	565 (7.8)	
1 time	953 (10.5)	104 (10.9)	
2 or 3 times	549 (6.1)	65 (11.8)	
4 times or more	277 (3.1)	59 (21.3)	
Experience of bullying			*p* < 0.0001
Never (0 days)	7312 (80.8)	527 (7.2)	
1 or 2 days	1251 (13.8)	156 (12.5)	
3 or more days	489 (5.4)	110 (22.5)	
Anxiety	358 (3.9)	89 (24.9)	*p* < 0.0001
Loneliness	583 (6.4)	137 (23.5)	*p* < 0.0001
Hunger	605 (6.7)	69 (11.4)	*p* < 0.05

*Health-risk behavior*			
Did not eat fruit	781 (8.6)	94 (12)	*p* < 0.0001
Current cigarette use	745 (8.2)	78 (10.5)	
Current alcohol use	239 (2.6)	48 (20.1)	*p* < 0.0001
Drunk from alcohol	219 (2.4)	39 (17.8)	*p* < 0.0001
Trouble from using alcohol	108 (1.2)	27 (25)	*p* < 0.0001
Ever use amphetamine	39 (0.4)	14 (35.9)	*p* < 0.0001
Sedentary behavior	1990 (22)	225 (11.30)	*p* < 0.0001

*Protective factors*			
Parental supervision	3815 (42.2)	251 (6.6)	*p* < 0.0001
Parental emotional support	3573 (39.5)	272 (8.8)	*p* < 0.0001
Parents know free time	3880 (42.9)	300 (7.7)	*p* < 0.0001
Number of close friends			
None (0)	377 (4.2)	91 (24.1)	*p* < 0.0001
1	1195 (13.2)	107 (8.9)	
2	1115 (12.3)	110 (9.9)	
3 or more	6365 (70.3)	485 (7.6)	

**Table 3:** Logistic regression analysis for factors associated with suicidal behavior.

Variables	Crude OR (95% CI)	Adjusted OR^*∗*^ (95% CI)
Sociodemographic		
Age group in year		—
13 or less	1 (reference)	
14-15	1.07 (0.91–1.26)	
16 or more	1.12 (0.90–1.38)	
Gender		
Male	1 (reference)	1 (reference)
Female	1.32 (1.13–1.53)	1.57 (1.33–1.86)

*Psychosocial factors*		
Missed class		
Never (0 days)	1 (reference)	1 (reference)
1 or 2 days	1.32 (1.11–1.57)	1.29 (1.08–1.55)
3 or more days	2.49 (1.85–3.33)	1.68 (1.21–2.33)
Physically attacked		
Never (0 times)	1 (reference)	1 (reference)
1 time	1.29 (1.04–1.61)	1.06 (0.84–1.34)
2 or 3 times	1.61 (1.32–1.97)	1.21 (0.97–1.52)
4 times or more	2.29 (1.83–2.85)	1.34 (1.02–1.74)
Physical fight		
Never (0 times)	1 (reference)	1 (reference)
1 time	1.45 (1.16–1.81)	1.25 (0.97–1.60)
2 or 3 times	1.59 (1.21–2.09)	1.11 (0.82–1.52)
4 times or more	3.21 (2.38–4.33)	1.93 (1.35–2.76)
Experience of bullying		
Never (0 days)	1 (reference)	1 (reference)
1 or 2 days	1.83 (1.51–2.21)	1.41 (1.15–1.73)
3 or more days	3.73 (2.96–4.70)	2.36 (1.82–3.06)
Anxiety	3.75 (2.91–4.83)	2.04 (1.53–2.73)
Loneliness	3.65 (2.97–4.50)	2.16 (1.71–2.73)
Hunger	1.37 (1.05–1.78)	1.08 (0.81–1.43)

*Health-risk behavior*		
Did not eat fruit	1.48 (1.17–1.86)	1.33 (1.04–1.70)
Current cigarette use	1.24 (0.97–1.58)	—
Current alcohol use	2.72 (1.96–3.76)	1.86 (1.17–2.96)
Drunk from alcohol	2.32 (1.62–3.30)	0.86 (0.49–1.50)
Trouble from using alcohol	3.55 (2.28–5.53)	1.40 (0.72 0 2.73)
Ever use amphetamine	5.91 (3.06–11.43)	1.54 (0.65–3.65)
Sedentary behavior	1.45 (1.23–1.71)	1.24 (1.04–1.48)

*Protective factors*		
Parental supervision	0.61 (0.52–0.71)	0.62 (0.52–0.74)
Parental emotional support	0.78 (0.67–0.91)	0.89 (0.75–1.06)
Parent know free time	0.79 (0.68–0.92)	0.94 (0.80–1.12)
Number of close friends		
None	1 (reference)	
1	0.30 (0.22–0.42)	0.35 (0.25–0.48)
2	0.34 (0.25–0.46)	0.38 (0.27–0.53)
3 or more	0.25 (0.20–0.33)	0.28 (0.22–0.37)

^*∗*^adjusted to other associated variables in the crude OR.

## Data Availability

We used already available secondary data from the World Health Organization (WHO) Global School-based Student Health Survey (GSHS), which was designed to collect data from school-aged adolescents in developing countries, including Bangladesh and Indonesia. The data that support the findings of this study are available on request from the corresponding author.

## Supplementary Materials

Supplementary Materials Supplementary file 1: the description of variables.
