# Supplementary material for: Rate and Associated Factors of Suicidal Behavior among Adolescents in Bangladesh and Indonesia: Global School‐Based Student Health Survey Data Analysis
Source: Scientifica (Cairo). 2022 Aug 5;2022:8625345. doi: 10.1155/2022/8625345 (PMC9410988; doi:10.1155/2022/8625345)
Supplement: Supplementary Materials — Supplementary file 1: the description of variables. [file 8625345.f1.docx]

Supplementary file 1: The description of variables

|  |  |  |
| --- | --- | --- |
| **Variables** | **Question** | **Response options (coding scheme)** |
|  | **Outcome Variable** |  |
| Suicide ideation | "During the past 12 months, did you ever seriously consider attempting suicide?" | "1=Yes, 2=No (coded 1=1, 2=0)" |
| Suicide plan | "During the past 12 months, did you make a plan about how you would attempt suicide?" | "1=Yes, 2=No (coded 1=1, 2=0)" |
| Suicide attempt | "During the past 12 months, how many time did you actually attempt suicide?" | “1=0 times to 5 = 6 or more times” (coded 1=0, 2-5=1) |
|  |  |  |
|  | **Demographic variables** |  |
| Age | “How old are you?” | “11 years old or younger to 18 years old or older” |
| Gender | “What is your sex?” | “Male, Female” |
|  |  |  |
|  | **Psychosocial factors** |  |
| Anxiety | “During the past 12 months, how often have you been so worried about something that you could not sleep at night?” | “1 = never to 5 = always (coded 1-3=0 and 4-5=1)” |
| Loneliness | “During the past 12 months, how often have you felt lonely?” | “1 = never to 5 = always (coded 1-3=0 and 4-5=1)” |
| Missed class | "During the past 30 days, on how many days did you miss classes or school without permission? | “1 = never to 5 = always (coded 1=0, 2=1 and 3-5=2)” |
| Physically attacked | “During the past 12 months, how many times were you physically attacked?” | “1=0 times to 8=12 or more times” (coded 1=0, 2=1, 3=2 and 4-8=3) |
| Physical fights | “During the past 12 months, how many times were you in a physical fight?” | “1=0 times to 8=12 or more times” (coded 1=0, 2=1, 3=2 and 4-8=3) |
| Experience of bullying | “During the past 30 days, on how many days were you bullied?” | “1=0 days to 7=All 30 days” (coded 1=0, 2=1 and 3-7=2) |
| Hunger | “During the past 30 days, how often did you go hungry because there was not enough food in your home?" | “1 = never to 5 = always (coded 1-3=0 and 4-5=1)” |
|  |  |  |
|  | **Health risk behaviour** |  |
| Did not eat fruit | “During the past 30 days, how many times per day did you usually eat fruit such as apples, oranges, and bananas?” | “1=I did not eat fruit during the past 30 days to 7=5 or more times per day (coded 1=1 and 2-7=0)" |
| Current cigarette use | “During the past 30 days, on how many days did you smoke cigarettes?" | “1=0 days to 7=All 30 days (coded 1=0 and 2-7=1)” |
| Current alcohol use | "During the past 30 days, on how many days did you have at least one drink containing alcohol?" | “1=0 days to 7=All 30 days (coded 1=0 and 2-7=1)” |
| Drunk from alcohol | "During your life, how many times did you drink so much alcohol that you were really drunk?" | "1 = 0 times to 4 = 10 or more times, (coded 1=0 and 2 - 4 = 1)" |
| Trouble from using alcohol | "During your life, how many times have you got into trouble with your family or friends, missed school, or got into fights, as a result of drinking alcohol?" | "1 = 0 times to 4 = 10 or more times, (coded 1=0 and 2 - 4 = 1)" |
| Ever use amphetamine | “During your life, how many times have you used amphetamines or methamphetamines?” | “1=0 times to 5=20 or more times (coded 1=0 and 2-5=1)” |
| Sedentary behaviour | “How much time do you spend during a typical or usual day sitting and watching television, playing computer games, talking with friends, or doing other sitting activities, such as using the computer or cell phone?” | “1=Less than 1 hour per day… 3= 3 to 4 hours per day …6=8 or more than 8 hours a day (coded 1-2=0 and 3-6=1) |
|  |  |  |
|  | **Protective Factors** |  |
| Parental supervision | “During the past 30 days, how often did your parents or guardians check to see if your homework was done?” | “1=never to 5=always (coded 1-3=0 and 4–5=1)” |
| Parental emotional support | “During the past 30 days, how often did your parents or guardians understand your problems and worries?” | “1=never to 5=always (coded 1-3=0 and 4–5=1)” |
| Parent know free time | “During the past 30 days, how often did your parents or guardians really know what you were doing with your free time?” | “1=never to 5=always (coded 1-3=0 and 4–5=1)” |
| Number of close friends | “How many close friends do you have?” | “1 = 0 to 4 = 3 or more (coded 1=0, 2=1, 3=2 and 4=3)” |
